# Prevalence, Treatment Patterns, and Outcomes of Individuals with *EGFR* Positive Metastatic Non-Small Cell Lung Cancer in a Canadian Real-World Setting: A Comparison of Exon 19 Deletion, L858R, and Exon 20 Insertion *EGFR* Mutation Carriers

**DOI:** 10.3390/curroncol29100567

**Published:** 2022-09-30

**Authors:** Dylan E. O’Sullivan, Tamer N. Jarada, Amman Yusuf, Leo (Xun Yang) Hu, Priyanka Gogna, Darren R. Brenner, Erica Abbie, Jennifer B. Rose, Kiefer Eaton, Julia Elia-Pacitti, Emmanuel M. Ewara, Aliyah Pabani, Winson Y. Cheung, Devon J. Boyne

**Affiliations:** 1 Department of Oncology, University of Calgary, Calgary, AB T2N 4N2, Canada; 2 Oncology Outcomes Initiative, University of Calgary, Calgary, AB T2N 4N2, Canada; 3 Public Health Sciences, Queen’s University, Kingston, Toronto, ON K7L 3N6, Canada; 4 Janssen Inc., Toronto, ON M3C 1L9, Canada

**Keywords:** non-small cell lung cancer, epidermal growth factor, genomic testing, healthcare resource use, real world evidence

## Abstract

Real-world evidence surrounding *EGFR* positive NSCLC patients in Canada is limited. Administrative databases in Alberta, Canada were used to evaluate *EGFR* testing and mutation prevalence in de novo metastatic NSCLC, as well as the characteristics, treatment patterns, and outcomes of individuals with Exon 19, L858R and Exon20ins mutations. Between 2013–2019, 2974 individuals underwent *EGFR* testing, of which 451 (15.2%) were *EGFR* positive. Among *EGFR* positive individuals, 221 (49.0%) had an Exon 19 mutation, 159 (35.3%) had an L858R mutation, and 18 (4%) had an Exon20ins mutation. The proportion of individuals who initiated 1L systemic therapy was 89.1% for Exon19, 85.5% for L858R, and 72.2% for Exon20ins carriers. The primary front-line systemic therapy was gefitinib or afatinib monotherapy for individuals with Exon 19 (93.4%) and L858R (94.1%) mutations versus platinum combination therapy for individuals with Exon20ins mutations (61.5%). The Exon20ins cohort had worse median overall survival from initiation of 1L systemic therapy (10.5 months [95% CI: 8.0-not estimable]) than the Exon19 (20.6 months [95% CI: 18.4–24.9]), and L858R cohorts (19.1 months [95% CI: 14.5–23.1]). These findings highlight that Exon20ins mutations represent a rare subset of NSCLC in which treatment options are limited and survival outcomes are worse relative to individuals with more common types of *EGFR* mutations.

## 1. Introduction

Lung cancer is the most commonly diagnosed cancer in Canada, with an estimated 30,000 new cases expected in 2022 [1]. Non-small cell lung cancer (NSCLC) accounts for approximately 85% of all lung cancer cases [2]. The epidermal growth factor receptor (EGFR) is involved in the regulation of both cell proliferation and apoptosis via signal transduction pathways [3]. The dysregulation of EGFR can lead to uncontrolled cell proliferation, angigenesis, and migration, which contribute to tumour growth and spread, and therefore presents as an important therapeutic target [3]. More than 60% of NSCLCs have been identified to have overexpression of *EGFR*, and in Canada, *EGFR* mutations are identified in approximately 15% of all lung cancer cases [3,4]. Testing for *EGFR* mutations in patients with NSCLC is the current standard of care in Canada since 2013, following the introduction of EGFR tyrosine kinase inhibitors (TKIs) in 2010 [5]. TKIs work to inhibit improper activity of EGFR, which is involved in both cell apoptosis and tumor progression [6].

The most common types of *EGFR* mutations are the exon 19 deletion (Exon19), and exon 21 codon 858 point mutation (L858R) [5]. *EGFR* exon 20 insertion mutations (Exon20ins) are rare compared with these common mutations [5,7], and have been associated with resistance to currently approved EGFR, TKIs and poorer clinical outcomes. Exon20ins comprise a diverse group of mutations, which makes them difficult to detect in NSCLC patients [7]. The increasing use of next-generation sequencing (NGS) technologies in oncology has allowed for improved detection of rarer mutations such as Exon20ins, subsequently leading to an increase in the clinical relevance of this mutation group in NSCLC patients [8,9]. Due to the lower response from all TKIs and worse prognoses in NSCLC with Exon20ins [7,10], identifying these mutations in NSCLC patients is critical to providing appropriate therapy and improving clinical outcomes [11]. NSCLC patients with Exon20ins have been the target for several ongoing clinical trials [12].

There is a lack of Canadian data within the *EGFR* positive NSCLC setting, particularly with respect to Exon20ins. The purpose of this real-world investigation was to characterize the rate of *EGFR* testing among individuals diagnosed with de novo metastatic NSCLC in Alberta, Canada between 2013–2019 and to describe the prevalence, treatment patterns, and outcomes of those who had Exon19, L858R, and Exon20ins mutations.

## 2. Materials and Methods

This was a longitudinal, retrospective, observational cohort study of adults (≥18 years of age) with newly (de novo) diagnosed metastatic NSCLC in Alberta, Canada. Since *EGFR* testing in Alberta began in 2013, analyses were restricted to individuals newly diagnosed between 2013–2019.

Individuals with NSCLC were identified using Alberta’s provincial cancer registry, with *EGFR* testing information obtained from the Northern and Southern Alberta Laboratory Testing Centres. *EGFR* mutation testing was completed with either PCR (Qiagen kit) or NGS (Agena MassArray kit). Additional databases were linked to measure baseline characteristics using a unique identifier, and included electronic medical records as well as the Discharge Abstract Database (DAD), the National Ambulatory Care Reporting System Database (NACRS), and the Practitioner Claims Database. Relevant patient information was also abstracted, by trained medical doctors, from the charts of individuals with an Exon20ins mutation.

Testing patterns, baseline patient and disease characteristics, treatment patterns, and health outcomes (overall survival (OS), time to next treatment (TTNT), and healthcare resource utilization (HCRU)) were evaluated. For individuals with Exon20ins mutations, progression free survival (PFS) was also examined using data extracted from the charts. Chi-square and analysis of variance (ANOVA) tests were used to compare baseline characteristics between patients with Exon19, L858R, and Exon20ins mutations. *EGFR* testing that occurred within a specific time window of 90 days after diagnosis was assessed. When estimating *EGFR* testing rates, analyses were restricted to individuals diagnosed prior to 30 September 2019, to allow for the 90-day time window from diagnosis to receipt of test results. Exploratory subgroup analyses for *EGFR* testing rates were also conducted by year of diagnosis, geography of residence (urban/rural), among those with non-squamous NSCLC, and among those referred to a medical oncologist.

Treatment patterns were identified using an algorithm adapted from a previous lung cancer study [13]. The start date of the line of therapy was defined as the earliest date of systemic treatment given on or after the date of initial diagnosis. All systemic agents received within 30 days of the start date were used to define the regimen. The start date of a subsequent line of therapy was defined as the earliest of the following two dates, if available: (a) date on which patient received any new systemic agent; or (b) date on which there was a gap equal to or greater than 90 days between successive systemic agents. This procedure was repeated to classify subsequent lines of therapy. The end date of each line of therapy was defined as the earliest of the following three dates: start date of the subsequent line of therapy, date of the last agent received within the line of therapy plus 21 days, and last known date of follow-up or death.

OS was defined as time from diagnosis or start of the current line of therapy until death of any cause, while TTNT was defined as time from the initiation of the current line of therapy until the initiation of a new line of therapy or death from any cause, whichever came first. PFS for Exon20ins patients was defined as time from diagnosis or start of the current line of therapy until disease progression (ascertained from chart review) or death. Survival curves and median time-to-event were estimated with the Kaplan–Meier method and differences in time-to-event outcomes by patient groups were tested with log-rank tests. Due to the descriptive nature of this study, there was no adjustment for confounding.

Healthcare resource utilization was examined from diagnosis within each year of follow-up. For each construct, we estimated the total number of events and the mean number of events per patient whereby the total number of events within each year of follow-up was divided by the number of patients alive at the start of each year of follow-up. The following constructs were examined: number of hospitalizations and days hospitalized, encounters with ambulatory care services, non-cancer practitioner encounters, cancer physician visits, days of radiation therapy, and cycles of systemic therapy. With respect to non-cancer practitioner claims, several claims within a single study were considered to constitute a single encounter. All analyses were conducted using the R computing framework (www.r-project.org, accessed on 7 February 2022).

## 3. Results

To assist with dissemination of the study findings we have included a plain language summary in the supplementary materials.

### 3.1. Study Population

A total of 6666 patients diagnosed with de novo metastatic NSCLC between 2013–2019 were identified.

### 3.2. EGFR Testing Patterns and Prevalence of EGFR Mutations

**Figure 1:**
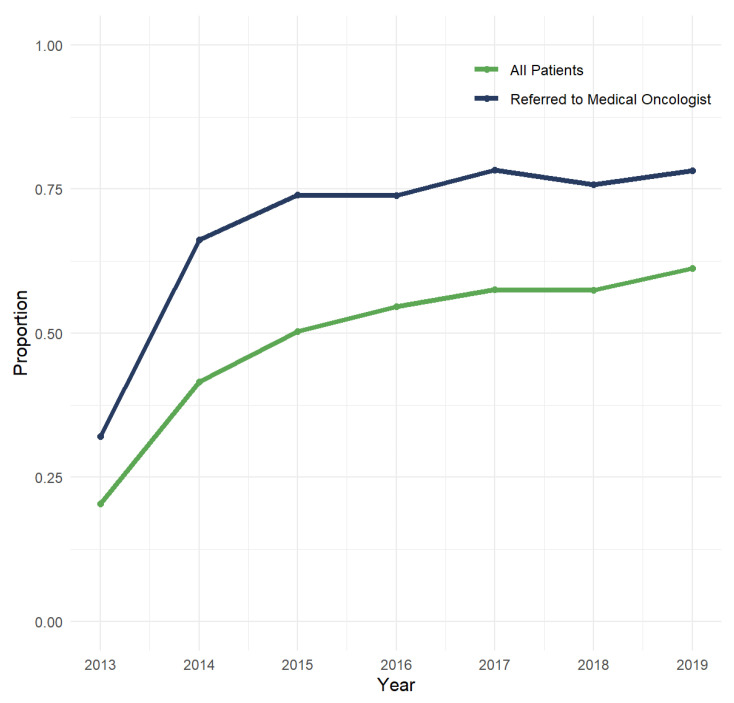
Annual rate of *EGFR* testing conducted within 90 days of diagnosis among individuals with de novo metastatic NSCLC.

Since 2013, annual *EGFR* testing rates have steadily risen from 20.4% in 2013 to 61.3% in 2019 (Figure 1). After restricting to 2017–2019, exploratory analyses showed higher testing rates among individuals with non-squamous NSCLC who were referred to a medical oncologist (88.3%) and that individuals who resided in rural areas had significantly lower testing rates (50.3%) compared to those in urban areas (60.2%; *p* < 0.001).

Among individuals who underwent *EGFR* testing between 2013 and 2019 and who had adequate DNA (*n* = 2974), 451 (15.2%) were *EGFR* positive. Out of the 451 patients with a detected *EGFR* mutation, 221 (49%) had an Exon19 mutation, 159 (35.3%) had an L858R mutation, 18 (4.0%) had an Exon20ins mutation, 31 (6.9%) had an Exon18 mutation, 19 (4.2%) had an Exon20del mutation, 14 (3.1%) had an L861Q mutation, and 3 (0.7%) had an EGFR mutation of uncertain location (note: 14 of the 451 (3.1%) individuals with *EGFR* mutations had two mutations; there were no instances of individuals simultaneously having any combination of Exon20ins, L858R, or Exon19, which are the focus of subsequent analyses).

### 3.3. Baseline Characteristics

At the time of initial diagnosis for individuals with an Exon19, L858R, or Exon20ins mutation (*n* = 398), the mean age was 66.7 years (SD: 13.6), 35.9% were male, 93% had an adenocarcinoma (versus 5% unknown and 2% other), 42% had 1+ Charlson comorbidities, 33.7% presented with 3+ metastatic sites, and 26.6% had brain metastasis (Table 1). The median time from diagnosis to *EGFR* test results was 18 days. There was a significant difference in age among the EGFR groups with Exon20ins patients being the youngest and L858R the oldest (*p* = 0.029). There was some evidence that L858R patients were less likely to be male (*p* = 0.056), while no significant differences were observed for the other baseline characteristics (Table 1).

### 3.4. Treatment Patterns

The median number of lines of therapy completed by patients was 2 lines for the Exon19 and L858R (IQR: 1 to 3) and 1 line for the Exon20ins group (IQR: 0.0 to 2). Of the 221 patients in the Exon19 mutation group, 197 (89.1%) initiated 1L systemic therapy, 129 (65.5%) initiated 2L, 81 (62.8%) initiated 3L, and 44 (54.3%) initiated 4L. Of the 159 patients in the L858R patient group, 136 (85.5%) initiated 1L, 81 (59.6%) initiated 2L, 41 (50.6%) initiated 3L, and 19 (46.3%) initiated 4L. Of the 18 patients in the Exon20ins patient group, 13 (72.2%) initiated 1L therapy, 8 (61.5%) initiated 2L therapy, and <5 (<27.8%) initiated 3L therapy.

Gefitinib monotherapy was the most commonly prescribed therapy in 1L for individuals with Exon19 (*n* = 129, 65.5%) or L858R (*n* = 96, 70.6%) mutations, followed by afatinib monotherapy (Exon 19: *n* = 55, 27.9%; L858R: *n* = 32, 23.5%). In contrast, the primary frontline therapy used in Exon20ins carriers was a platinum combination therapy (*n* = 8, 61.5%); none of the Exon20ins patients received gefitinib monotherapy. Other therapies included afatinib monotherapy, chemotherapy plus an EGFR inhibitor, or other immunotherapy (*n* < 5).

Among individuals with Exon19 or L858R mutations who initiated 2L (*n* = 210), gefitinib monotherapy was the most common treatment (*n* = 84, 40.0%), followed by osimertinib monotherapy (*n* = 52, 24.8%). In the 3L setting (*n* = 122), osimertinib monotherapy was the most common treatment modality (*n* = 43, 35.2%) followed by gefitinib monotherapy (*n* = 27, 22.1%). There was inadequate sample size to examine treatment patterns in the 2L+ setting for individuals with Exon20ins.

### 3.5. Time-to-Event Outcomes

**Figure 2:**
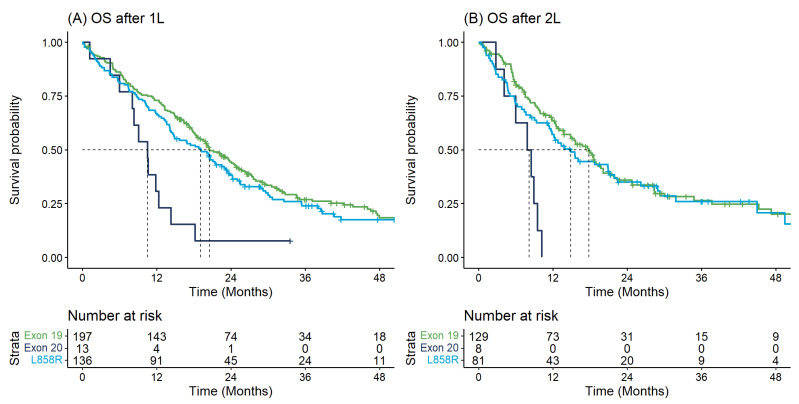
Overall survival for first and second-line therapy: (**A**) Overall survival from start of first-line therapy by *EGFR* mutation type; (**B**) Overall survival from start of second-line therapy by *EGFR* mutation type.

**Figure 3:**
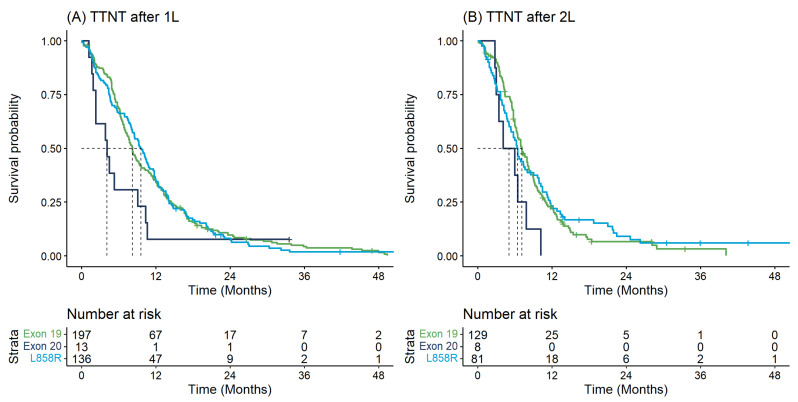
Time to next treatment for first and second-line therapy: (**A**) Time to next treatment from start of first-line therapy by *EGFR* mutation type; (**B**) Time to next treatment from start of second-line therapy by *EGFR* mutation type.

Table 2 describes the key time-to-event outcomes. OS from diagnosis differed significantly by *EGFR* mutation type (log-rank *p*-value = 0.018) whereby individuals with Exon20ins had shorter median OS from diagnosis (11.2 months [95% CI: 9.5–24.4]) versus those with Exon19 (20.8 months [95% CI: 18.2–24.4]) or L858R (15.7 months [95% CI: 14.1–21.9]) (Table 2). These findings were similar when examining survival outcomes from initiation of 1L or 2L therapy and with respect to TTNT (Table 2, Figure 2 and Figure 3).

Median PFS for individuals with Exon20ins was 7.0 months since diagnosis (95% CI: 3.3–15.1), 4.4 months since initiation of 1L (95% CI: 1.5-not estimable), and 3.3 months since initiation of 2L (95% CI: 2.2-not estimable).

To assist with dissemination of the study findings and synthesis of the results we have included a plain language summary and graphical abstract in the supplementary materials.

### 3.6. Healthcare Resource Utilization

Healthcare resource utilization was largely comparable between different *EGFR* mutation types, with higher rates of utilization in the first year of diagnosis and declining rates in subsequent years (Table 3).

## 4. Discussion

Herein, we present real-world evidence pertaining to *EGFR* testing rates and the characteristics, treatment patterns, and outcomes of *EGFR* mutation carriers in the Canadian setting. In this study we found that testing for *EGFR* mutations in NSCLC patients has increased over time. While the rate of *EGFR* testing was 61.3% in 2019, we found that this lower rate of testing was primarily attributable to lower rates of testing among individuals with squamous disease and those who were not referred to a medical oncologist. Among those who underwent testing, turnaround time was relatively short (18 days). Future qualitative research is needed to help identify potential barriers to and reasons for a lack of *EGFR* testing.

Among those who underwent testing, the overall prevalence of *EGFR* mutations was 15.2%, with Exon20ins representing less than 1% of NSCLC patients. The prevalence of *EGFR* mutations was in line with previous estimates for Canada [4] but is lower than that observed in many treatment and referral based studies [14] which have been shown to overestimate *EGFR* prevalence in the total population [15]. A previous Canadian study, which focused on the molecular profiling of mutations in NSCLC, found a lower prevalence of *EGFR* mutations (6.9%) in their cohort, but the cohort was primarily composed of early stage cases [16]. To our knowledge there are no recent population-based studies in Canada on *EGFR* prevalence in advanced or metastatic NSCLC.

We found that individuals with Exon20ins tended to have worse survival and complete fewer lines of therapy compared to those with the more common Exon 19 and L858R mutations, which may be attributable to the lack of targeted therapies available to Exon20ins carriers. A recent study from the United States using the Flatiron Database quantified the prevalence, treatment patterns, and survival of advanced NSCLC patients with an *EGFR* mutation, with a focus on Exon20ins mutations [17]. The prevalence of an Exon20ins mutation was 6.4% among those with an *EGFR* positive test, which is comparable to our estimate of 4.0% [17]. Similar to our investigation, patients with an Exon20ins mutation most commonly received platinum-based chemotherapy and tended to have worse survival outcomes compared to other *EGFR* mutations [17].

Other studies have also investigated various aspects of Exon20ins patient groups. A recent systematic review found the prevalence of the Exon20ins mutation ranged from 0.1–4% of all NSCLC cases, and 1–12% of all *EGFR* mutations [18]. Treatments assigned to Exon20ins cohorts have been varied in 1L, and have included afatinib, gefitinib, erlotinib, osimertinib, platinum-based therapies, immune-oncology agents IOs, and other TKIs [17,19,20,21,22,23,24]. Despite the variation, platinum-based chemotherapies were the most common treatment for those patients, which is similar to what was observed in this study [17]. Median OS among studies identified in the systematic review for Exon20ins cohorts have ranged from 7.1 to 18.2 months, which is comparable to our finding of 10.5 months from diagnosis [18]. The PFS result from our study (4.4 months from 1L) was also comparable to previous papers, with PFS ranging from 1.9 to 6.4 [17,19,20,21,22,23,24].

To our knowledge, this investigation is among the first to quantify healthcare resource utilization differences among *EGFR* positive NSCLC cases. A recent systematic review, completed in 2021, aimed to search for studies on HCRU and did not identify any [18]. We searched for studies published since the search date cut-off used in the review and similarly did not find any additional papers. In this study we observed that healthcare resource utilization was largely comparable between different *EGFR* mutation types, with higher rates of utilization in the first year of diagnosis and declining rates in subsequent years for all *EGFR* mutation types.

An important limitation of this analysis includes the lack of precision for some estimates due to small sample sizes, particularly for the Exon20ins cohort considering the rarity of this mutation. It is also possible that some Exon20ins mutation patients were missed due to the type of testing used. PCR testing was primarily used to identify *EGFR* mutations in this cohort. However, this methodology may have missed certain types of mutations. Specifically, single-gene PCR testing is known to be inferior to other methods, such as multi-plex NGS, for identifying subtypes of *EGFR* mutations [18,25]. Data for a subset of baseline covariates of interest could not be assessed, such as smoking history or performance status. We used an algorithm to determine lines of therapy in patients, and some misclassification may have occurred using this method. Finally, the treatment patterns reported on were all prior to the COVID-19 pandemic and covered a limited time for capturing provincial utilization of osimertinib, which was funded in Alberta in 2018 for use in 2nd or later lines for patients with T790M mutations and in 2020 for use in 1st line for individuals with Exon19 deletion or L858R mutations. As such, the treatment patterns reported in this paper may not represent more current treatment practices for individuals diagnosed post-2019, particularly for Exon20ins patient where the introduction of additional EGFR targeted therapies such as amivantamab and mobocertinibin are anticipated in the near future. Finally, statements on comparative efficacy of therapies cannot be made on the basis of these results given the descriptive nature of the study.

The primary strength of this investigation was our ability to leverage a large, representative, population-based dataset to gather real-world evidence on *EGFR* testing patterns as well as the characteristics, treatment patterns, and outcomes of individuals who were *EGFR* positive. The leveraging of these data was particularly helpful with respect to the identification of individuals with Exon20ins given the rarity of this mutation and associated challenges of recruiting such individuals into a prospective study. To our knowledge, this is the largest and most current Canadian study on this topic. Lastly, our reliance on administrative data allowed us to objectively examine overall survival with an adequate follow-up duration and little risk of attrition bias.

## 5. Conclusions

Within the Canadian setting, the rate of *EGFR* mutations in metastatic NSCLC are comparable to those published in other regions at 15.2%. These findings confirm the rarity of Exon20ins mutations, with a prevalence of less than 1% among NSCLC patients who undergo *EGFR* testing. Rates of *EGFR* testing have increased over time, and were particularly high among those who were referred to a medical oncologist and who had non-squamous disease. Individuals with Exon20ins tended to have worse survival and complete fewer lines of therapy compared to those with the more common Exon 19 and L858R mutations. These findings highlight that Exon20ins mutations are exceedingly rare in NSCLC and are associated with worse overall survival and fewer treatment options compared to the more common Exon 19 and L858R *EGFR* mutations.

## Figures and Tables

**Table 1:** Baseline characteristics of *EGFR*-mutated metastatic NSCLC.

Variable	Overall *n* = 398	Exon 19 *n* = 221	Exon 20 *n* = 18	L858R *n* = 159	*p*-Value
Age at Diagnosis (mean (SD))	66.7 (13.6)	65.3 (13.9)	63.9 (15.2)	68.9 (12.6)	0.029
Diagnosed 2016–2019 (%)	252 (63.3)	144 (65.2)	12 (66.7)	96 (60.4)	0.606
Male (%)	143 (35.9)	89 (40.3)	8 (44.4)	46 (28.9)	0.056
1+ Charlson Comorbidities (%)	167 (42.0)	94 (42.5)	6 (33.3)	67 (42.1)	0.748
Adenocarcinoma (%)	370 (93.0)	208 (94.1)	18 (100.0)	144 (90.6)	0.201
No. of Metastatic Sites at Diagnosis (%)					0.777
1	152 (38.2)	82 (37.1)	6 (33.3)	64 (40.3)	
2	110 (27.6)	63 (28.5)	6 (33.3)	41 (25.8)	
3+	133 (33.4)	73 (33.0)	6 (33.3)	54 (34.0)	
Missing	3 (0.8)	3 (1.4)	0 (0.0)	0 (0.0)	
Brain Metastasis at Diagnosis (%)	106 (26.6)	57 (25.8)	3 (16.7)	46 (28.9)	0.490
Time to First *EGFR* Test, Days (median [IQR])	18.0 [14.0, 26.0]	18.0 [14.0, 25.0]	16.0 [14.0, 26.3]	19.0 [14.0, 26.0]	0.772

Abbreviations: IQR = interquartile range; No. = number; SD = standard deviation.

**Table 2:** Time-to-event outcomes of *EGFR*-mutated metastatic NSCLC patients by *EGFR* mutation type and line of therapy.

Variable	*N*	Median (Months)	1-Year (%)	2-Year (%)
		(95% CI)	(95% CI)	(95% CI)
Overall Survival from Diagnosis				
Exon 19	221	20.84 (18.25–24.39)	69.1 (63.3–75.5)	43.6 (37.5–50.8)
L858R	159	15.68 (14.07–21.90)	62.0 (54.9–70.1)	37.6 (30.8–46.1)
Exon 20	18	11.24 (9.47–24.43)	44.4 (26.5–74.5)	22.2 (9.4–52.7)
Overall Survival from 1L				
Exon 19	197	20.55 (18.41–24.89)	73.0 (67.0–79.5)	44.4 (37.8–52.1)
L858R	136	19.10 (14.53–23.11)	66.9 (59.5–75.3)	38.2 (30.8–47.5)
Exon 20	13	10.52 (8.02–NA)	30.8 (13.6–69.5)	7.7 (1.2–50.6)
Overall Survival from 2L				
Exon 19	129	17.79 (13.74–20.09)	63.5 (55.5–72.6)	35.9 (27.8–46.3)
L858R	81	14.89 (11.41–21.90)	57.2 (47.2–69.2)	35.1 (25.6–48.2)
Exon 20	8	8.14 (5.98–NA)	0.0 (NA–NA)	0.0 (NA–NA)
Overall Survival from 3L				
Exon 19	81	13.22 (10.55–22.16)	54.3 (44.0–67.0)	35.6 (25.5–49.5)
L858R	41	18.71 (10.16–NA)	55.7 (41.8–74.2)	39.4 (25.1–61.9)
Exon 20	<5			
TTNT from 1L				
Exon 19	197	8.19 (7.36–9.30)	34.2 (28.2–41.5)	9.6 (6.2–14.9)
L858R	136	9.58 (8.22–10.95)	34.6 (27.4–43.6)	8.1 (4.5–14.5)
Exon 20	13	4.11 (2.30–NA)	7.7 (1.2–50.6)	7.7 (1.2–50.6)
TTNT from 2L				
Exon 19	129	7.13 (6.28–8.22)	22.0 (15.7–30.7)	6.6 (3.2–13.7)
L858R	81	6.44 (5.33–8.98)	23.3 (15.6–34.8)	9.1 (4.4–19.0)
Exon 20	8	5.05 (3.42–NA)	0.0 (NA–NA)	0.0 (NA–NA)
TTNT from 3L				
Exon 19	81	5.75 (5.10–7.76)	15.7 (9.2–26.9)	4.5 (1.3–15.9)
L858R	41	6.51 (5.75–8.88)	24.9 (14.5–42.9)	9.3 (3.3–26.6)
Exon 20	<5			

Abbreviations: CI = confidence interval; L = line of therapy.

**Table 3:** Mean (standard deviation) healthcare resource utilization per patient per year from diagnosis among *EGFR*-mutated metastatic NSCLC cases by *EGFR* mutation type.

EGFR Strata	Construct	Outcome	Year 1	Year 2	Year 3	Year 4	Year 5
Exon 19	N	N	221	152	85	39	23
	Hospital	Hospitalizations	0.94 (1.13)	0.74 (1.03)	0.49 (0.93)	0.38 (0.71)	0.57 (1.04)
		Days Hospitalized	11.52 (23.71)	6.75 (13.50)	4.09 (8.53)	9.69 (29.60)	5.65 (14.66)
	Ambulatory	All Encounters	9.76 (7.21)	8.25 (23.98)	4.67 (5.65)	4.10 (3.64)	4.96 (5.57)
		Emergency	1.65 (1.86)	1.35 (1.83)	1.15 (2.48)	0.82 (1.10)	1.00 (1.51)
		Non-Emergency	8.12 (6.79)	6.90 (23.94)	3.52 (4.41)	3.28 (3.38)	3.96 (4.63)
	Cancer Physician	All Visits	10.27 (6.65)	8.93 (7.93)	7.46 (7.26)	7.33 (7.09)	8.30 (9.08)
		Medical Oncologist	7.79 (5.54)	7.23 (6.69)	6.32 (6.74)	6.00 (6.32)	7.35 (7.98)
		Radiation Oncologist	2.01 (3.04)	1.13 (2.14)	0.76 (1.55)	Suppressed	Suppressed
		Other Cancer Physician	0.47 (1.52)	0.57 (1.62)	0.38 (1.36)	<10	<10
	Non-Cancer Practitioner	Claims	53.56 (46.72)	35.15 (36.54)	26.80 (28.67)	27.18 (42.82)	28.61 (47.02)
		Costs	4117.06 (3521.70)	2413.29 (2511.54)	1958.29 (2223.42)	1625.30 (2081.08)	2570.54 (4278.09)
		Encounters	28.82 (23.86)	18.12 (15.62)	14.84 (13.04)	14.54 (18.00)	14.91 (16.98)
	Radiation Therapy	Days	6.41 (9.49)	3.37 (6.88)	2.28 (5.45)	2.87 (6.80)	1.70 (5.49)
	Chemotherapy Cycles	Cycles	7.43 (5.15)	5.41 (5.32)	4.41 (4.66)	3.30 (3.44)	4.55 (4.10)
Exon 20	N	N	18	8	<10	<10	<10
	Hospital	Hospitalizations	0.94 (0.73)	0.50 (0.76)			
		Days Hospitalized	9.89 (10.62)	8.12 (16.27)			
	Ambulatory	All Encounters	9.56 (7.06)	3.38 (3.96)			
		Emergency	2.50 (2.83)	1.12 (1.36)			
		Non-Emergency	7.06 (6.17)	2.25 (2.92)			
	Cancer Physician	All Visits	15.72 (10.97)	3.50 (3.42)			
		Medical Oncologist	10.78 (9.39)	2.75 (3.06)			
		Radiation Oncologist	3.00 (4.64)	0.25 (0.46)			
		Other Cancer Physician	1.94 (3.78)	0.50 (1.07)			
	Non-Cancer Practitioner	Claims	47.44 (44.44)	27.00 (29.39)			
		Costs	3448.43 (2288.78)	1562.61 (1771.47)			
		Encounters	26.00 (21.48)	14.00 (15.76)			
	Radiation Therapy	Days	10.39 (13.86)	1.00 (2.45)			
	Chemotherapy Cycles	Cycles	8.62 (4.17)	1.17 (1.94)			
L858R	N	N	159	98	55	27	12
	Hospital	Hospitalizations	1.08 (1.23)	0.85 (1.26)	0.76 (1.25)	<10	<10
		Days Hospitalized	13.11 (21.63)	8.83 (15.23)	10.71 (23.83)	3.74 (11.74)	12.42 (24.69)
	Ambulatory	All Encounters	9.55 (8.62)	6.45 (6.29)	4.13 (6.10)	1.89 (2.81)	4.00 (6.05)
		Emergency	2.21 (3.90)	1.47 (2.01)	1.09 (2.03)	0.52 (0.75)	1.17 (1.85)
		Non-Emergency	7.35 (7.01)	4.98 (5.57)	3.04 (5.15)	1.37 (2.53)	2.83 (4.32)
	Cancer Physician	All Visits	10.88 (8.07)	9.70 (8.81)	7.60 (8.25)	7.41 (6.88)	8.83 (6.55)
		Medical Oncologist	8.21 (6.81)	8.07 (8.12)	6.87 (7.74)	6.41 (6.46)	7.75 (6.06)
		Radiation Oncologist	2.16 (2.97)	1.12 (1.97)	0.33 (0.79)	Suppressed	Suppressed
		Other Cancer Physician	0.52 (1.53)	0.51 (1.52)	0.40 (1.23)	<10	<10
	Non-Cancer Practitioner	Claims	59.50 (54.13)	42.64 (47.93)	35.38 (55.42)	21.22 (37.21)	45.75 (73.76)
		Costs	4885.42 (4806.53)	2984.61 (3384.87)	2433.08 (3906.33)	1971.04 (4076.41)	2884.29 (4868.36)
		Encounters	30.46 (24.59)	23.53 (22.59)	18.13 (21.75)	10.44 (15.02)	21.00 (30.93)
	Radiation Therapy	Days	7.53 (9.91)	2.57 (5.88)	1.11 (3.59)	2.63 (6.51)	2.42 (4.46)
	Chemotherapy Cycles	Cycles	10.85 (31.35)	7.04 (17.01)	5.48 (12.50)	3.04 (4.21)	2.83 (3.27)

## Data Availability

Aggregate-level data presented in this study are available on request from the corresponding author. Individual-level data are not publicly available due to Canadian data privacy laws governing personal health information.

## Supplementary Materials

The following are available online at https://www.mdpi.com/article/10.3390/curroncol29100567/s1, File S1, Plain Language Summary.
