# Supplementary material for: Prevalence, Treatment Patterns, and Outcomes of Individuals with EGFR Positive Metastatic Non-Small Cell Lung Cancer in a Canadian Real-World Setting: A Comparison of Exon 19 Deletion, L858R, and Exon 20 Insertion EGFR Mutation Carriers
Source: Curr Oncol. 2022 Sep 30;29(10):7198–208. doi: 10.3390/curroncol29100567 (PMC9600059; doi:10.3390/curroncol29100567)
Supplement: Supplementary file 1 [file curroncol-29-00567-s001.zip › curroncol-1876861-supplement.pdf]

Plain Language Summary

Prevalence, Treatment Patterns, and Outcomes of Individuals with EGFR Positive Metastatic Non-Small Cell Lung Cancer in a Canadian Real-World Setting: A Comparison of Exon 19 Deletion, L858R, and Exon 20 Insertion EGFR Mutation Carriers

WHY THIS STUDY IS NEEDED

Lung cancer is the most common cancer in Canada and leads to almost 25% of all cancer related deaths in Canada[1]. There are many different types of lung cancer. The most common type of lung cancer is non-small cell lung cancer (NSCLC) which makes up about 85% of all lung cancer cases[2]. Many patients with NSCLC have a mutation, which is a type of change or damage to the DNA in the lungs. These mutations are often what leads to a cancer developing.

We identify mutations through special tests (sometimes called biomarker tests) after a patient has surgery. There are three types of mutations that we focused on in this study:

- Epidermal Growth Factor Receptor (EGFR) Exon 19 deletion mutation (Exon19)
- EGFR Exon 21 codon 858 point mutation (L858R)
- EGFR Exon 20 insertion mutation (Exon20ins)

There is a lot we do not know about how these mutations affect patients' survival. This study aimed to answer questions about patient outcomes related to these different types of mutations in Alberta.

WHAT THE STUDY DID

Real world evidence is a type of research that uses health information that doctors collect during treatment visits. Doctors enter patient health information into many different databases. The information from these different databases is combined together by researchers, and helps to study how patients are treated and how they do. This information gives researchers a big picture understanding of if there might be better ways to treat patients.

This study looked at health information for patients 18 and older, diagnosed with NSCLC in Alberta, Canada between 2013-2019. For all patients in this study, it was the first time they had cancer, and the cancer had already spread to other parts of the body.

In this study, we wanted to understand how doctors treat patients with the following questions:

- who gets tested for these mutations?
- when do they get tested?

---

[1] Brenner et al. Projected Estimates of Cancer in Canada 2022. CMAJ. https://www.cmaj.ca/content/194/17/E601

[2] Canadian Cancer Statistics Advisory Committee. Canadian Cancer Statistics: A 2020 special report on lung cancer. Toronto, ON: Canadian Cancer Society; 2020. Available at: cancer.ca/Canadian-Cancer-Statistics-2020-EN

- are there differences in patient characteristics in these testing groups and do these patients live longer?
- does the type of mutation a patient has affect how long they live?
- are there differences in health care use between mutation types?

WHAT THE STUDY FOUND

Between 2013-2019, there were 6,666 patients diagnosed with NSCLC whose cancer metastasized, which means it had already spread to other parts of the body. For these patients, this was their first time being diagnosed with cancer.

There was an increase in mutation testing from 2013-2019 which led to more mutations being detected. This is what we found:

- On average, about 60% of patients with NSCLC are tested for mutations. Patients are more likely to have mutation tests if they see a cancer doctor, and if they live in a big city. About 15% of patients who receive testing will test positive for one of these types of mutation.
- If a patient has a mutation test, results are usually available about 18 days after their cancer diagnosis.
- Depending on the type of mutation a patient has, different treatments may work better, and patients may live longer. This study found that patients with Exon 19 & L858R mutations lived longer than those with Exon20ins mutation.
- If a doctor gives a patient a treatment specific to their mutation, the patient may live longer.
- The frequency of doctors visits and hospitalizations were similar among all mutations types

WHY THIS MATTERS TO PATIENTS AND DOCTORS

Testing for mutations is important because mutations affect treatment and survival. If doctors know the type of mutation their patient has, they can make better treatment decisions. Better treatment decisions may lead to patients living longer.

More patients are now being tested for genetic mutations in cancer than ever before. Of those tested, about 15% of patients have mutations. Some patients are not tested for mutations. We need more research to understand why that is.

Different mutations may require different treatments in order for patients to live longer. Patients with different types of mutations could benefit from new treatments in the future.

ACKNOWLEDGMENTS

This plain language summary was reviewed by the Oncology Outcomes Patient and Family Advisory Council, Lung Health Foundation, and Lung Cancer Canada.
